# Supplementary material for: Total Metabolic Tumor Volume on 18F-FDG PET/CT Is a Useful Prognostic Biomarker for Patients with Extensive Small-Cell Lung Cancer Undergoing First-Line Chemo-Immunotherapy
Source: Cancers (Basel). 2023 Apr 10;15(8):2223. doi: 10.3390/cancers15082223 (PMC10136504; doi:10.3390/cancers15082223)

Total metabolic tumor volume on 18F-FDG PET/CT is a useful prognostic biomarker for patients with extensive small cell lung cancer undergoing first-line chemo-immunotherapy

SUPPLEMENT

SUPPLEMENTAL TABLES 2

Table S1: Area under the curve (AUC) for the prediction of PFS and OS at 6, 12, and 18 months in the chemo-immunotherapy (CIT) and chemotherapy (CT) groups based on total metabolic tumor volume (TMTV) and derived neutrophil-to-liver ratio (dNLR) 2

Table S2: Tests for the additional prognostic value of TMTV and dNLR in the chemo-immunotherapy group 3

Table S3: Estimated Overall Survival (OS) probability at 6, 12, and 18 months in the chemo-immunotherapy (CIT) cohort based on total metabolic tumor volume (TMTV) and liver metastases (LM) status 4

Table S4: Estimated Overall Survival (OS) probability at 6, 12, and 18 months in the chemotherapy (CT) cohort based on total metabolic tumor volume (TMTV) and liver metastases (LM) status..5

SUPPLEMENTAL FIGURES 6

Figure S1: Predictiveness curves for TMTV (A) and Tumor SUVmax (B) for 6-month-PFS...6

Figure S2: Relationships between FDG uptake of tumor (tumor SUVmax), metabolic tumor burden (TMTV) and clinic-biological parameters (age, leukocytes, neutrophils, lymphocytes and dNLR) evaluated by Spearman's correlation coefficients 7

Figure S3: Kaplan-Meier curves of Overall Survival (OS) (A) and Progression-Free Survival (PFS) (B) according to the derived Neutrophil-to-Lymphocyte Ratio (dNLR) in the chemotherapy (CT) cohort 8

Figure S4: Kaplan-Meier curves of Progression-Free Survival (PFS) based on total metabolic tumor volume (TMTV) and liver metastases (LM) status in the chemo-immunotherapy (CIT) cohort (A) and in the chemotherapy (CT) cohort (B) 9

<u>Table S1:</u> Area under the curve (AUC) for the prediction of PFS and OS at 6, 12, and 18 months in the chemo-immunotherapy (CIT) and chemotherapy (CT) groups based on total metabolic tumor volume (TMTV) and derived neutrophil-to-liver ratio (dNLR).

**AUC (95%CI)**	**PFS**			**OS**		
	6 months	12 months	18 months	6 months	12 months	18 months
**CHEMO-IMMUNOTHERAPY GROUP**						
dNLR	0.55 (0.35-0.74)	0.70 (0.44-0.95)	0.66 (0.33-1.00)	0.85 (0.61-1.00)	0.72 (0.54-0.90)	0.62 (0.40-0.82)
TMTV	0.59 (0.40-0.79)	0.63 (0.35-0.90)	0.62 (0.45-0.78)	0.88 (0.73-1.00)	0.61 (0.42-0.80)	0.63 (0.44-0.83)
**CHEMOTHERAPY GROUP**						
dNLR	0.61 (0.39-0.82)	0.58 (0.38-0.78)	0.58 (0.39-0.79)	0.67 (0.41-0.88)	0.70 (0.51-0.89)	0.77 (0.60-0.94)
TMTV	0.63 (0.43-0.83)	0.61 (0.40-0.81)	0.61 (0.41-0.82)	0.64 (0.40-0.87)	0.56 (0.36-0.77)	0.63 (0.36-0.91)

<u>Abbreviations</u>: progression-free survival (PFS), overall survival (OS), total metabolic tumor volume (TMTV), derived neutrophil-to-lymphocyte ratio (dNLR).

Table S2: Tests for the additional prognostic value of TMTV and dNLR in the chemo-immunotherapy group.

Legend: Likelihood ratio test and p values were generated comparing multivariable Cox proportional hazard models with and without the addition of TMTV (> 241 cm3 versus ≤ 241 cm3) or with and without dNLR (> 3 versus ≤ 3) in order to test for its additional prognostic value. The multivariable Cox model was generated with backward elimination for Cox regression. The multivariable model for OS included the following clinical variables: age (≥ 70 vs< 70 years), dNLR (>3 vs ≤ 3), liver metastases (yers vs no) and TMTV (> 241 cm3 versus ≤ 241 cm3). The multivariable model for PFS included the following clinical variables: dNLR (>3 vs ≤ 3) and TMTV (> 241 cm3 versus ≤ 241 cm3).

	CHEMO-IMMUNOTHERAPY GROUP			
	PFS		OS	
**Cox model variables**	LR $\chi 2$	LR p value	LR $\chi 2$	LR p value
Multivariable model (with TMTV and dNLR)	6.9	-	12.9	-
Multivariable model without TMTV	3.5	0.06	9.0	0.04
Multivariable model without dNLR	3.8	0.08	8.3	0.03

Abbreviations: likelihood ratio (LR), progression-free survival (PFS), overall survival (OS), total metabolic tumor volume (TMTV), derived neutrophil-to-lymphocyte ratio (dNLR).

<u>Table S3:</u> Estimated Overall Survival (OS) probability at 6, 12, and 18 months in the chemo-immunotherapy (CIT) cohort based on total metabolic tumor volume (TMTV) and liver metastases (LM) status.

Strata	Time (months)	n risk	n event	Survival probability [95% CI]
High risk	6	6	2	0.63 [0.50–1.00]
	12	2	6	0.12 [0.02–0.78]
	18	0	8	0.00 [NA–NA]
Intermediate risk	6	10	0	0.90 [0.73–1.00]
	12	5	5	0.51 [0.26–1.00]
	18	3	2	0.26 [0.08–0.84]
Low risk	6	18	0	0.94 [0.84–1.00]
	12	11	7	0.60 [0.40–0.92]
	18	5	13	0.33 [0.16–0.68]

<u>Abbreviations:</u> confidence interval (CI), not applicable (NA).

<u>Table S4:</u> Estimated Overall Survival (OS) probability at 6, 12, and 18 months in the chemotherapy (CT) cohort based on total metabolic tumor volume (TMTV) and liver metastases (LM) status.

Strata	Time (months)	n risk	n event	Survival probability [95% CI]
High risk	6	6	1	0.71 [0.45–1.00]
	12	3	4	0.29 [0.09–0.92]
	18	1	6	0.14 [0.02–0.88]
Intermediate risk	6	9	1	0.80 [0.59–1.00]
	12	4	6	0.30 [0.12–0.77]
	18	1	9	0.10 [0.02–0.64]
Low risk	6	12	3	0.73 [0.54–0.99]
	12	8	7	0.47 [0.27–0.80]
	18	4	11	0.27 [0.12–0.62]

Abbreviations: confidence interval (CI).

Figure S1: Predictiveness curves for TMTV (A) and Tumor SUVmax (B) for 6-month-PFS.

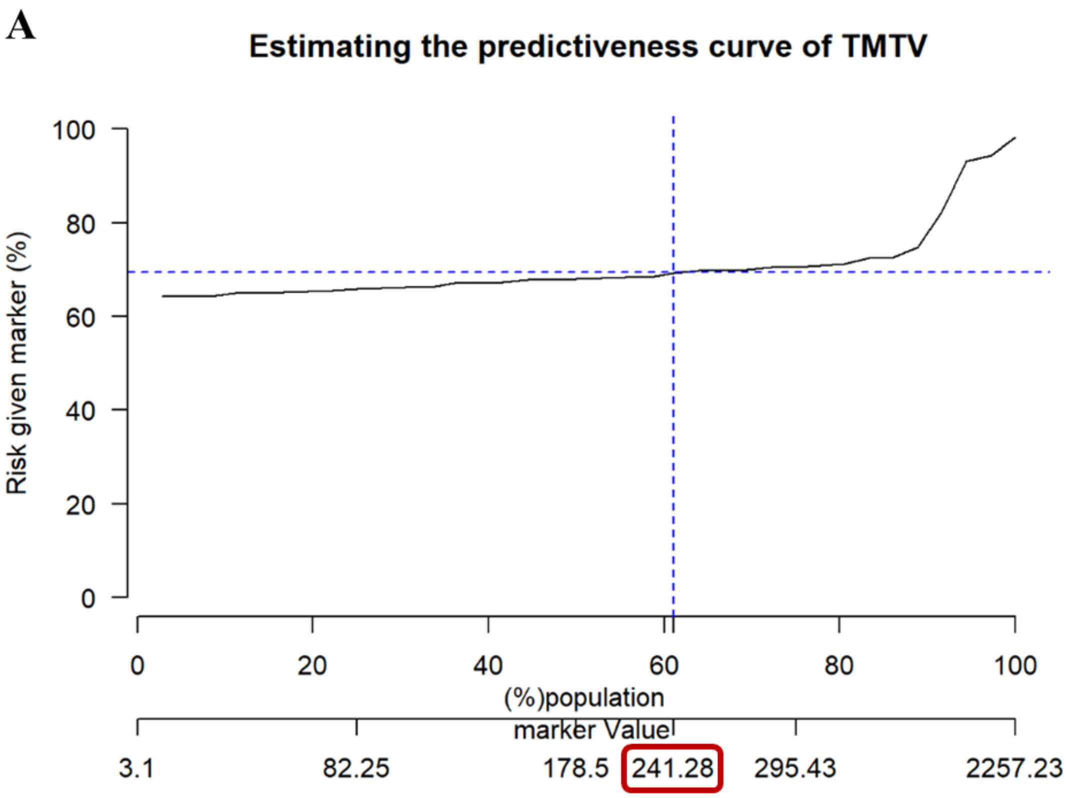

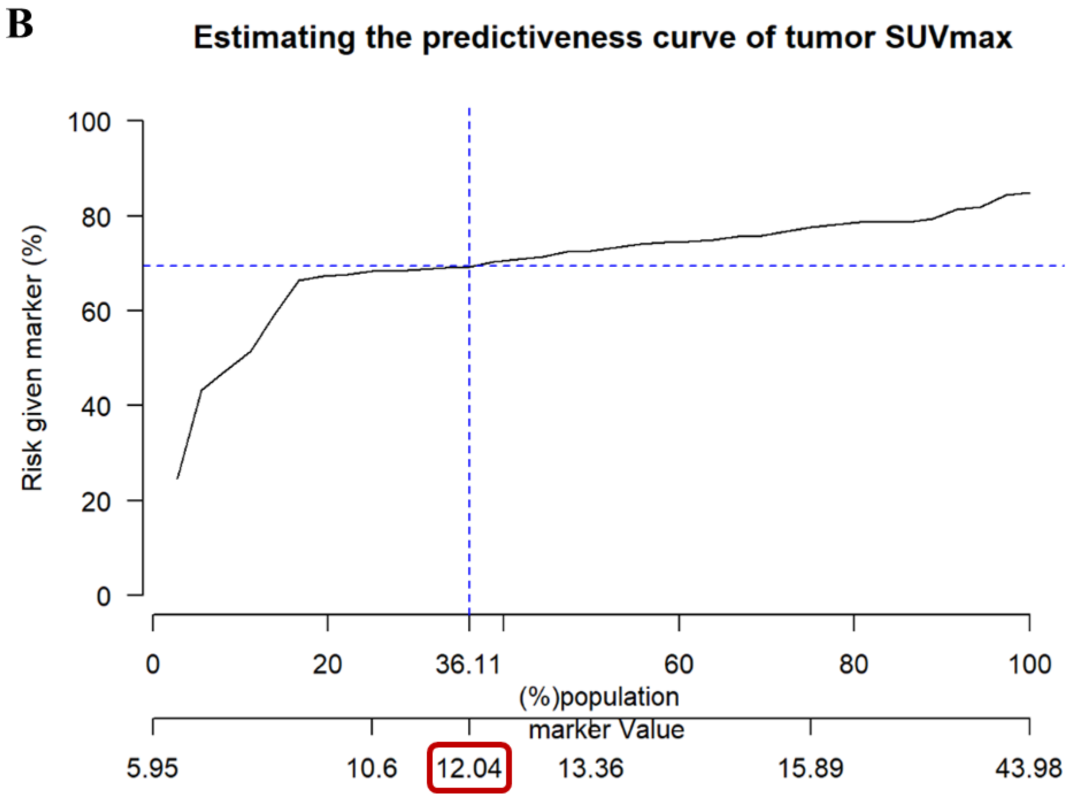

Figure S2: Relationships between FDG uptake of tumor (tumor SUVmax), metabolic tumor burden (TMTV) and clinic-biological parameters (age, leukocytes, neutrophils, lymphocytes and dNLR) evaluated by Spearman's correlation coefficients.

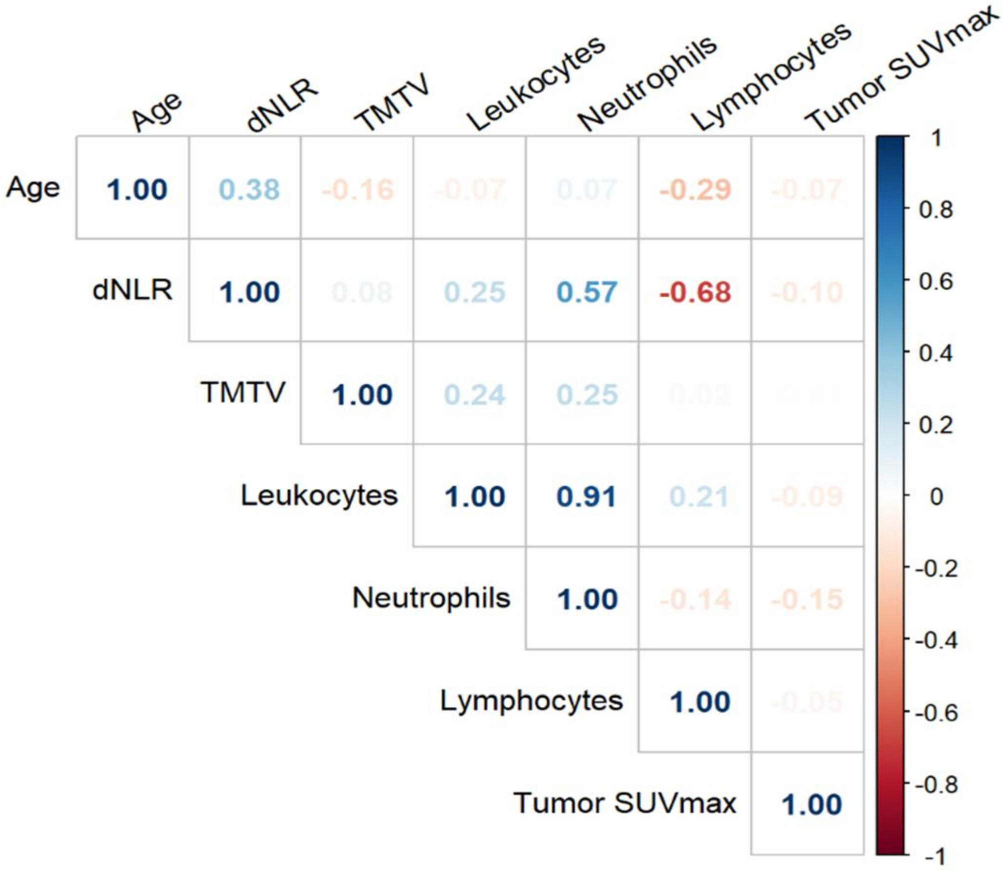

Figure S3: Kaplan-Meier curves of Overall Survival (OS) (A) and Progression-Free Survival (PFS) (B) according to the derived Neutrophil-to-Lymphocyte Ratio (dNLR) in the chemotherapy (CT) cohort.

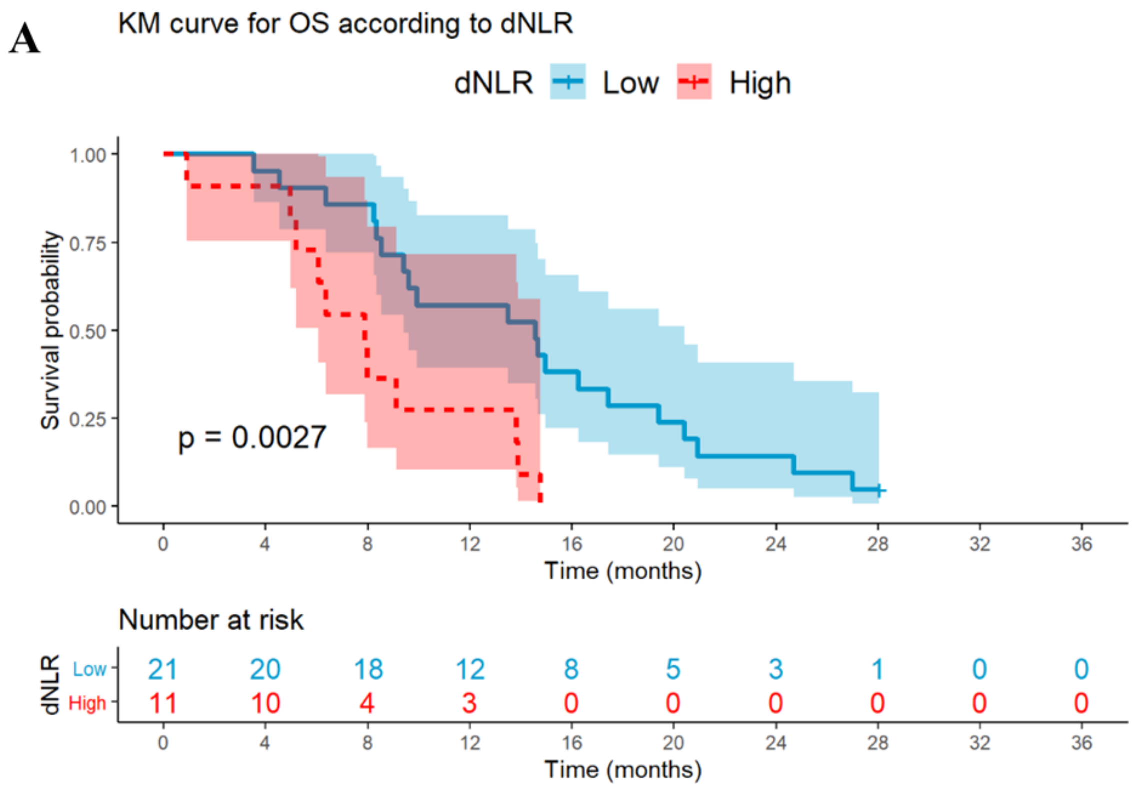

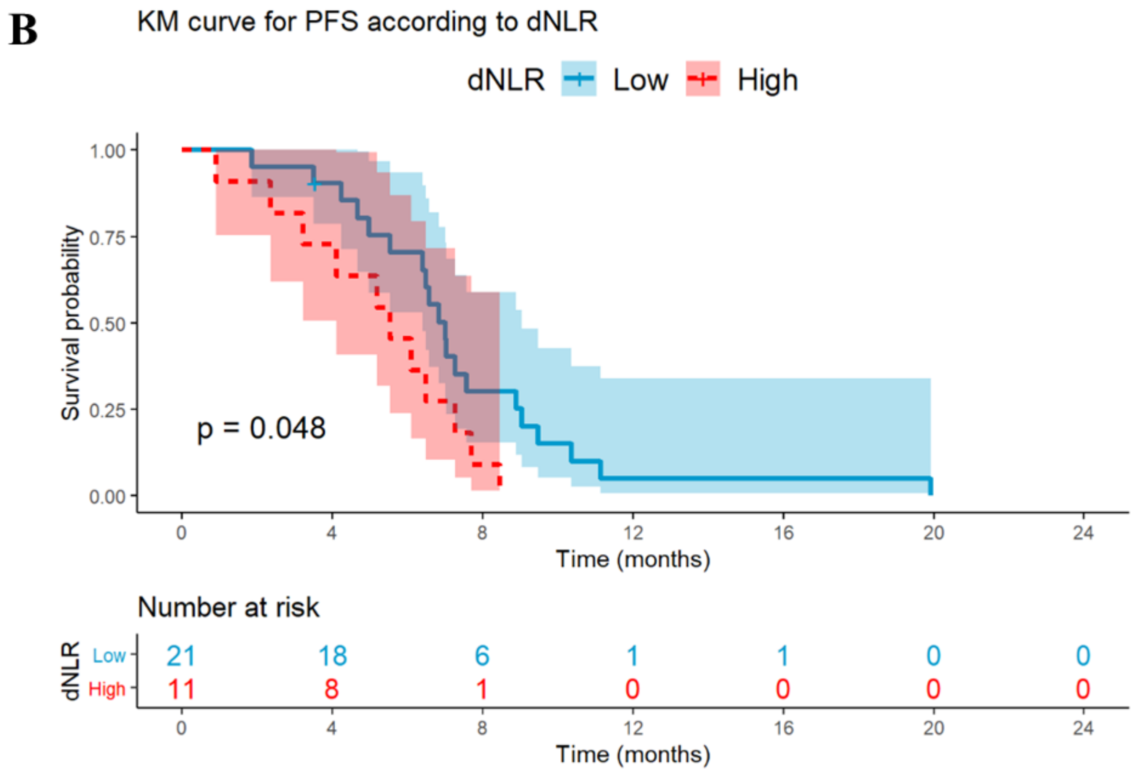

<u>Figure S4:</u> Kaplan-Meier curves of Progression-Free Survival (PFS) based on total metabolic tumor volume (TMTV) and liver metastases (LM) status in the chemo-immunotherapy (CIT) cohort (A) and in the chemotherapy (CT) cohort (B).

A

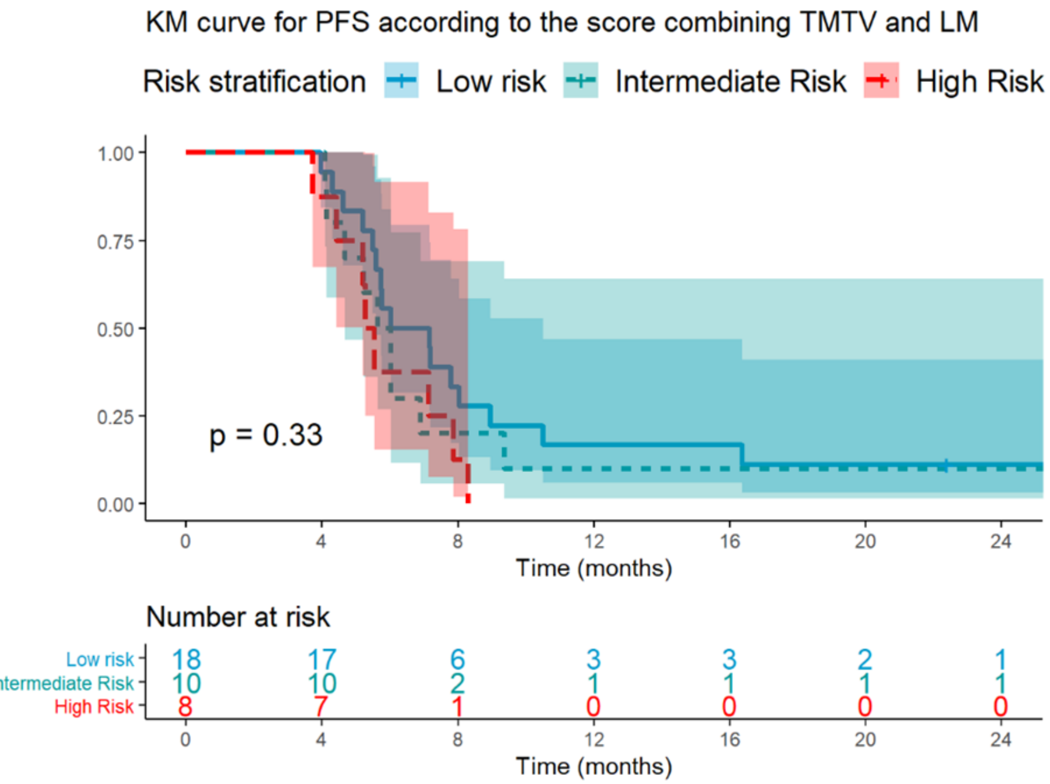

B

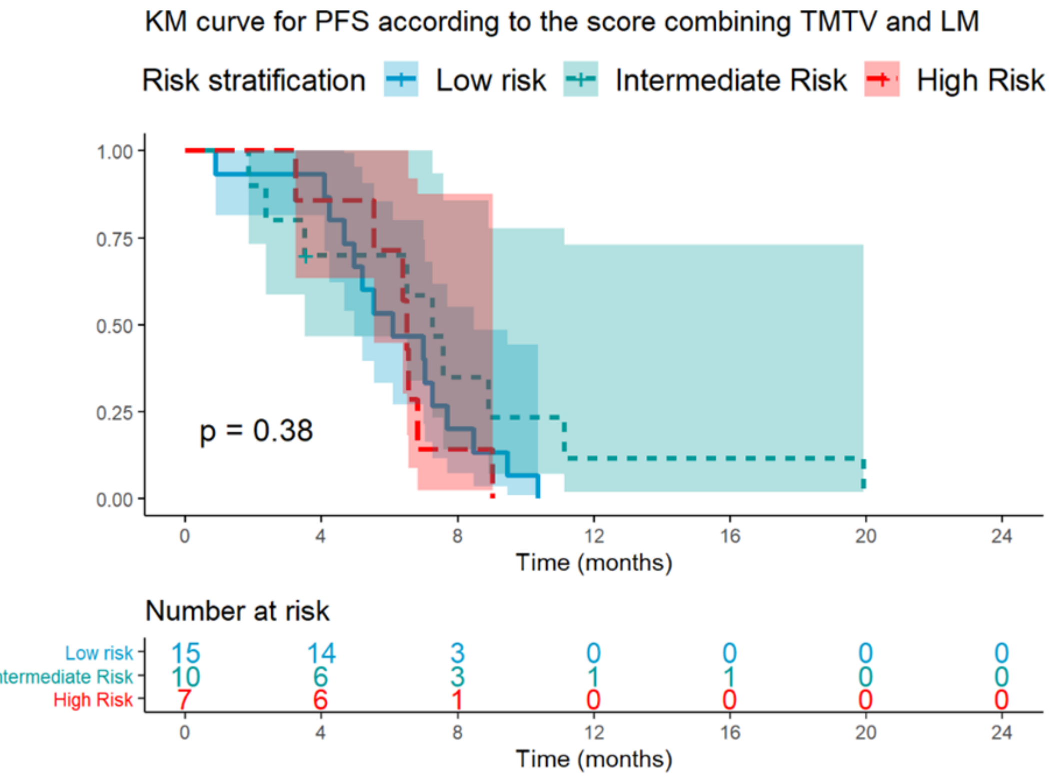

Supplement: Supplementary file 1 [file cancers-15-02223-s001.zip › cancers-2288551-supplementary.pdf]
